# Developmental Neurotoxicity of Alcohol and Anesthetic Drugs Is Augmented by Co-Exposure to Caffeine

**DOI:** 10.3390/brainsci3031128

**Published:** 2013-07-30

**Authors:** Carla M. Yuede, John W. Olney, Catherine E. Creeley

**Affiliations:** 1 Department of Neurology, Washington University School of Medicine, St. Louis, MO 63110, USA; E-Mail: yuedec@neuro.wustl.edu; 2 Department of Psychiatry, Washington University School of Medicine, St. Louis, MO 63110, USA; E-Mail: olneyj@psychiatry.wustl.edu

**Keywords:** CAF, alcohol, anesthetic drugs, neuroapoptosis, developing brain

## Abstract

Anesthetic and anti-epileptic drugs used in pediatric and obstetric medicine and several drugs, including alcohol, that are abused by pregnant women, trigger widespread neuroapoptosis in the developing brain of several animal species, including non-human primates. Caffeine (CAF) is often administered to premature infants to stimulate respiration, and these infants are also exposed simultaneously to anesthetic drugs for procedural sedation and/or surgical procedures. Pregnant women who abuse alcohol or other apoptogenic drugs also may heavily consume CAF. We administered CAF to infant mice alone or in combination with alcohol, phencyclidine, diazepam, midazolam, ketamine, or isoflurane, which are drugs of abuse and/or drugs frequently used in pediatric medicine, and found that CAF weakly triggers neuroapoptosis by itself and markedly potentiates the neuroapoptogenic action of each of these other drugs. Exposure of infant mice to CAF + phencyclidine resulted in long-term impairment in behavioral domains relevant to attention deficit/hyperactivity disorder, whereas exposure to CAF + diazepam resulted in long-term learning/memory impairment. At doses used in these experiments, these behavioral impairments either did not occur or were substantially less pronounced in mice exposed to CAF alone or to phencyclidine or diazepam alone. CAF currently enjoys the reputation of being highly beneficial and safe for use in neonatal medicine. Our data suggest the need to consider whether CAF may have harmful as well as beneficial effects on the developing brain, and the need for research aimed at understanding the full advantage of its beneficial effects while avoiding its potentially harmful effects.

## 1. Introduction

Acute exposure of the developing animal brain to several classes of drugs, including those that block NMDA glutamate receptors, those that activate GABA_A_ receptors, and alcohol (which has both NMDA antagonist and GABA-mimetic properties), triggers widespread apoptotic death of neurons [1,2,3,4,5,6,7,8,9,10,11,12,13,14,15,16,17,18,19,20,21,22,23,24,25,26,27]. Numerous animal species, including non-human primates [28,29,30,31,32,33,34,35,36,37,38,39,40] are susceptible. Early exposure to these agents is associated with long-term neurobehavioral disturbances in both rodents [6,25,41,42,43,44,45] and non-human primates [37]. The window of peak vulnerability coincides with the developmental period of rapid synaptogenesis [1,2], also known as the brain growth spurt period, which in mice and rats occurs primarily during the first 2 weeks after birth, but in humans extends from about mid-gestation to several years after birth [46]. 

Included among agents that have neuroapoptogenic activity are some that may be abused by pregnant mothers [alcohol, phencyclidine (PCP), ketamine, barbiturates, benzodiazepines], and many that are used in obstetric and pediatric medicine. Of major concern are alcohol and pediatric drugs in the sedative-anesthetic and anti-epileptic categories, because millions of human fetuses or infants are exposed to these agents every year throughout the world. Evidence implicating agents in these categories as human neuropathogens includes the following: (1) Exposure of the human fetus to alcohol is well known to cause neuropathological changes in the developing brain and long-term neurobehavioral disturbances (termed fetal alcohol spectrum disorders, FASD) [47,48,49]; (2) Results of recent multicenter studies [50,51,52] indicates that children who were exposed *in utero* to the anti-epileptic drug (AED), valproate, during the third trimester of pregnancy have a 9–12 point deficit in IQ, and third trimester exposure to other AEDs (carbamazepine, lamotrigine, phenytoin) was associated with significant impairment in verbal communication skills; (3) Several recently reported studies [53,54,55,56,57,58,59] by independent research groups document that exposure of human infants to brief anesthesia significantly increases risk for long-term neurocognitive impairment in domains relevant to attention deficit/hyperactivity disorder (AD/HD) and/or a learning disability.

Pre-term infants are often exposed to multiple drugs in the neonatal intensive care unit (NICU), and the interactions between these agents have not been adequately studied. Because premature infants have frequent and repeated apnea and bradycardia spells, it is common practice to administer CAF to these infants in high doses to stimulate respiration and prevent spells of apnea [60,61]. When used in this context, CAF is considered safe and highly beneficial [62,63]. In fact, there is evidence that CAF can protect against hypoxia-induced brain damage in infant rats [64], and may reduce the incidence of cerebral palsy when administered to premature human infants [65]. However, there is also evidence that in the infant animal brain CAF may have neurotoxic properties. Kang *et al*. [66] reported that administration of CAF to 7 day old rats (50 mg/kg every 8 h × 3 = a total dose of 150 mg/kg) caused apoptotic neurodegeneration in several brain regions. Evidence that CAF is protective against hypoxia-induced brain damage, but can promote apoptotic neurodegeneration is not necessarily contradictory, in that hypoxic brain damage is mediated by an excitotoxic mechanism, and it has been demonstrated that excitotoxic and apoptotic neurodegeneration are two separate and distinct forms of cell death [67]. 

Premature infants who are exposed to high doses of CAF to stimulate respiration are also frequently exposed to sedative/anesthetic drugs (procedural sedation) on a repetitive or continuous basis, sometimes for days or weeks. They also may be exposed acutely to deep anesthesia for surgical procedures, in which case CAF is sometimes co-administered to counteract anesthesia-induced respiratory depression [60]. It is not known how CAF may interact with these anesthetic drugs at a cellular level in the developing brain, or whether such interaction is beneficial or detrimental. However, if CAF has pro-apoptotic properties [66], administering it together with anesthetic drugs that also have pro-apoptotic properties could hypothetically be detrimental. To test this hypothesis, we administered CAF alone or together with various drugs that have known apoptogenic properties to infant mice, and examined the brains several hours later for evidence of acute neuroapoptosis. We also conducted experiments aimed at determining how it may influence long-term neurobehavioral outcome, if infant mice exposed to an NMDA antagonist or a GABA agonist drug are also exposed simultaneously to CAF. 

## 2. Experimental Procedures

### 2.1. Subjects

All animal procedures were conducted in accordance with guidelines developed by the National Academy of Science and were approved by the Washington University Animal Care Committee. All of the subjects for these experiments were 4 day old (P4) ICR white mice. 

### 2.2. Blood CAF Levels

In planning these experiments, we first sought to determine what dose(s) of CAF should be administered to infant mice in order to induce clinically relevant CAF blood levels. In pediatric medicine blood levels of CAF in the range of 6 to 50 μg/mL are considered safe and therapeutic for premature infants [62]. Blood levels on the high side of this dose range have been documented due to extremely heavy consumption of CAF in pregnancy [68]. The phenomenon under study is an acute reaction—drug-induced developmental neuroapoptosis—that is triggered by a single dose of the apoptogenic agent. In the pediatric NICU, CAF is administered semi-chronically over a period of days or weeks. An initial bolus dose is given which causes high blood levels acutely that taper gradually and are supplemented by daily maintenance doses to keep the blood concentration at a desired steady-state level. In pilot experiments, we attempted to simulate the acute phase of this type of treatment regimen by administering a single subcutaneous (sc) dose of CAF (free base, Sigma-Aldrich Inc., St. Louis, MO, USA) to 4 day old infant mice at 40 or 80 mg/kg and found that this produced blood CAF levels at 6, 12 and 24 h of 12, 8.9, 2.7 or 65, 43 and 15 μg/mL, respectively. The mean CAF blood level over a 24 h period for 40 and 80 mg/kg doses was 7.4 and 38 μg/mL, respectively. Since these values are within the range (6–50 μg/mL) considered safe for premature human infants [62], we elected to use 40 and 80 mg/kg as our low and high doses respectively. 

### 2.3. Individual Experiments

#### 2.3.1. Experiment #1—Apoptogenic Action of CAF + Alcohol

In our first experiment, P4 mice (*n* ≥ 6 per group) were treated with saline (control group), or CAF (40 or 80 mg/kg), or alcohol (2.5 g/kg), or with CAF + alcohol at the same doses of CAF and alcohol. Six hours following drug administration all pups were deeply anesthetized, perfused with fixative (4% paraformaldehyde in Tris buffer), and their brains prepared for histological evaluation by methods described below. The dose of alcohol used in this experiment is quite low compared to doses in the range of 5 g/kg typically used by fetal alcohol researchers to study the toxic effects of alcohol on the developing brain [69]. The doses of CAF used are lower than those used by Kang *et al*. [66] in their original demonstration that CAF has apoptogenic properties. As indicated above, at 40 mg/kg and 80 mg/kg, CAF produces blood levels in the infant mouse that are in the low range and high range, respectively, of blood levels considered safe and therapeutic for human premature infants [62].

#### 2.3.2. Experiment #2—Apoptogenic Action of CAF + NMDA antagonists

Alcohol has both NMDA antagonist and GABAmimetic properties, so next we tested the effects of CAF in combination with drugs that have only NMDA antagonist or only GABAmimetic (see Section 2.3.3 below) properties. For the NMDA antagonist experiments we elected to study two agents, phencyclidine (PCP) and ketamine. Both of these NMDA antagonists have anesthetic properties and are sometimes abused by pregnant women. Many decades ago, PCP was approved by FDA as a dissociative anesthetic for use in human medicine, but it was soon withdrawn because of powerful psychotomimetic side effects. PCP is currently recognized as a drug of abuse (Angel Dust) that frequently causes acute schizophrenia-like psychotic reactions and also can trigger violent behaviors. Ketamine is a structural analog of PCP which has similar but milder side effects and is frequently used in pediatric medicine for induction of anesthesia, or as a procedural sedative. For the PCP experiments, P4 infant mice (*n* ≥ 6 per group) were treated subcutaneously with saline (control group), or CAF (80 mg/kg), or PCP (25 mg/kg), or with CAF + PCP at the same doses. For the ketamine experiments, P4 infant mice (*n* ≥ 6 per group) were treated subcutaneously with saline (control group), or CAF (80 mg/kg), or ketamine (40 mg/kg at time zero and 30 mg/kg 2 h later, or CAF + ketamine at the same doses. The doses of both PCP and ketamine are sub-anesthetic but deeply sedating. Ketamine was administered as an initial bolus followed by a subsequent maintenance dose because it has a short half-life. Six hours following drug administration all pups were deeply anesthetized, perfused with fixative (4% paraformaldehyde in Tris buffer), and their brains prepared for histological evaluation by methods described below.

#### 2.3.3. Experiment #3—Apoptogenic Action of CAF + GABAmimetics

To test the apoptogenic action of CAF + GABAmimetic agents, we chose diazepam and isoflurane. Diazepam is a prototypic member of the benzodiazepine class that acts at GABA_A_ receptors to enhance the inhibitory action of GABA. Benzodiazepines are the agents most frequently used in neonatal intensive care units for procedural sedation to keep infants in a state of reduced awareness, reduced sensitivity to pain, and reduced motor activity while diagnostic or therapeutic procedures are being performed. Diazepam is also used in pediatric medicine as an antiepileptic to arrest seizure activity, and is an anxiolytic drug that is sometimes abused by pregnant women. Human infants sometimes require surgery for life threatening conditions and, therefore, must be exposed acutely to anesthetic drugs at doses sufficient to render them unconscious and insentient to pain. Diazepam may be used for induction, and then isoflurane, or a similar halogenated ether (sevoflurane, desflurane), is routinely used to maintain a deep surgical plane of anesthesia for major surgical procedures lasting several or more hours. The anesthetic action of isoflurane is thought to be due primarily to an action at GABA_A_ receptors, although it may act by other mechanisms as well.

For the diazepam experiments, P4 infant mice (*n* ≥ 6 per group) were treated subcutaneously with saline (control group), or CAF (80 mg/kg), or diazepam (10 mg/kg) or with CAF + diazepam at the same doses. This dose of diazepam is sub-anesthetic but heavily sedating for the infant mouse. For the isoflurane experiments, P4 infant mice (*n* ≥ 6 per group) were exposed to saline in room air (control group), or CAF (80 mg/kg), or to isoflurane (2% concentration × 2 h duration), or to CAF + isoflurane. The concentration of isoflurane required to fully anesthetize an infant mouse is 2.7% [70], but this concentration suppresses respiration severely and causes a high mortality rate if respiratory support procedures are not being employed. Because of the small size of infant mice it is not feasible to intubate them or perform effective respiratory control procedures. To work around this problem, we limited the duration of anesthesia to 2 h, and the concentration of isoflurane to 2%, which provides moderate but not full anesthesia. Six hours following administration of drugs or saline, all pups were deeply anesthetized, perfused with fixative (4% paraformaldehyde in Tris buffer), and their brains prepared for histological evaluation by methods described below.

#### 2.3.4. Experiment #4—Long-Term Neurobehavioral Effects of CAF + NMDA antagonist or GABAmimetic

In light of recent evidence that brief exposure of human infants to general anesthesia is associated with increased risk for long-term neurocognitive disturbances [53,54,55,56,57,58,59] we conducted an experiment aimed at determining whether CAF can increase the potential of an NMDA antagonist or GABAmimetic drug to cause long-term neurobehavioral disturbances. For these experiments, we subjected P4 infant mice (n ≥ 10 per group) to treatment with PCP (10 mg/kg) or diazepam (10 mg/kg), either with or without co-administration of CAF. We originally intended to use the same dose of PCP (25 mg/kg) that was used in Section 2.3.2 above, but we found that mouse pups are very slow in recovering from this dose and this caused a high rate of maternal neglect and/or cannibalism. Therefore, a lower dose was used for these long-term neurobehavioral studies. Following treatment with saline, CAF, PCP, diazepam, or CAF + PCP or CAF + diazepam, the pups were kept warm in individual boxes containing bedding from the maternal nest for several hours until they recovered from anesthesia, then were returned to the maternal nest for long-term survival and neurobehavioral testing. At 21 days of age, pups were weaned and group-housed for behavioral testing beginning at P23. All mice were given a battery of sensorimotor tests to evaluate effects of the drug treatment on any aspects of balance, coordination or strength that may influence performance on the behavioral tests. Reactivity and habituation to handling for each animal was evaluated over 3 days by three independent raters using a scale from 1 to 5, with 1 being little or no movement and 5 being extreme reactivity including biting the handler or vocalization. Locomotor activity, rearing and exploratory behavior was assessed in activity chambers (Hamilton-Kinder) between P26 and P28. Learning and memory performance was measured from P28 to P45 using cued, place and probe versions of the Morris Water Maze similar to previously described methods [45]. The resident-intruder test was used to measure aggressive behaviors in adult male mice from P50 to P60. Male mice were individually housed for 1 week prior to testing and bedding was not changed for 3 days before introduction of the intruder mouse. Unfamiliar male C57/BL6 mice of the same age were used as intruders. A different, unfamiliar mouse was placed in the home cage of the test mouse on 3 consecutive days, and interactions were recorded and scored for a 10 min period using Stopwatch software (Emory University, GA, USA). Frequency, duration and latency for fighting, biting, pawing, following and alone time were scored for each session. 

Statistical analyses of the behavioral data were conducted using analysis of variance models (ANOVA), one-way ANOVA for sensorimotor and resident-intruder tests and Repeated Measures ANOVA for activity, learning and memory, reactivity to handling tests. Bonferroni correction was used for all *post hoc* comparisons (Statistica, StatSoft, Inc., Tulsa, OK, USA). 

### 2.4. Histopathology

In the original studies characterizing the apoptogenic properties of alcohol and anesthetic drugs [1,2,3,4,5,6,7,8,9,10,11,12], the cell death process was documented by TUNEL staining (non-specific marker for apoptosis), DeOlmos cupric silver staining (marker for cell death) and immunohistochemical staining for activated caspase 3 (AC3), a relatively specific marker for apoptosis. In addition, a detailed electron microscopic evaluation [1,2,4,6,7,10,12] revealed that the dying cells displayed all of the classical ultrastructural changes characteristic of apoptotic cell death, as originally described by Wyllie *et al*. [71]. In studies examining gene-regulated biochemical pathways, it was found that the cell death process involves translocation of Bax protein to mitochondrial membranes where it disrupts membrane permeability, allowing extra-mitochondrial leakage of cytochrome c, followed by a sequence of changes culminating in activation of caspase-3 [15,20,21]. Commitment to cell death occurs prior to the AC3 step [21]; therefore, immunohistochemical detection of neurons positive for AC3 has become recognized as a reliable means of mapping and quantifying dying cells that have already progressed beyond the point of cell death commitment. An additional feature of AC3 making it the method of choice for studying this cell death process is that in the early stages of cell death it stains the entire cell body and its processes, which facilitates identification of cell type, and in more advanced stages it reveals degenerative morphological changes (shrinkage, fragmentation, condensation) which allows one to trace the steps in the degenerative process. 

For the above reasons, AC3 immunohistochemistry was chosen as the method for mapping and quantifying the cell death process in the present study. Six hours after initiation of drug exposure, the pups were deeply anesthetized with pentobarbital and perfused with fixative (4% paraformaldehyde in Tris buffer) through the left cardiac ventricle and ascending aorta. The brains were removed from the skull and immersed in the perfusion fixative overnight in the refrigerator, then serially sectioned by vibratome in the sagittal plane and stained immunohistochemically for activated caspase-3 (AC3) by methods previously described [20,21]. Briefly, vibratome sections (70 µm thick) were washed in 0.01 M phosphate-buffered saline (PBS), quenched for 10 min in a solution of methanol containing 3% hydrogen peroxide, then incubated for 1 h in blocking solution (2% BSA/0.2% milk/0.1% Triton X-100 in PBS), followed by incubation overnight in rabbit anti-active caspase-3 antiserum (D175, Cell Signaling Technology, Beverly, MA, USA) diluted 1:1000 in blocking solution. Following incubation with D175 primary antibody, the sections were incubated for 1 h in secondary antibody (goat anti-rabbit 1:200 in blocking solution), and then reacted in the dark with ABC reagents (standard Vectastain ABC Elite Kit, Vector Labs, Burlingame, CA, USA) for 1 h. The sections were then washed 3 times with PBS, and incubated with VIP reagent (Vector VIP substrate kit for peroxidase, Vector Labs, Burlingame, CA, USA) to develop a purple color.

### 2.5. Quantitative Cell Counts

AC3-stained sagittal sections chosen at 0.5 mm intervals from the midline to lateral edge of the hemi-brain were imaged and quantitatively evaluated with the help of a stereology system consisting of the following components: Stereo Investigator (MicroBrightField, Inc., Colchester, VT, USA) on a Pentium III PC, connected to a Prior Optiscan motorized stage (ES103 XYZ system, Prior Scientific Inc., Rockland, MA, USA) mounted on a Nikon Labophot-2 microscope. The boundaries of each section were traced into the PC and from the tracings Stereo Investigator calculated the area in each section. AC3-positive neurons with visible processes were all counted. The population estimator function of Stereo Investigator was used to mark each profile while it was counted to ensure that no profile would be missed or counted twice. The counts from each section of a given brain were summed and divided by the tissue volume (total area times thickness of the sections) from which the counts were obtained to yield an estimate of the density of AC3-positive profiles (number per mm^3^). The counts were performed in a blinded manner in which the treatment condition was unknown to the person doing the counting. 

### 2.6. Statistical Methods

Statistical analysis of the neuroapoptosis density count data was performed by ANOVA followed by *post hoc* Bonferroni multiple comparisons test, using Prism 4.0b software (GraphPad Software Inc., San Diego, CA, USA). All data are expressed as Mean ± S.E.M. The probability level for significance was set at *p* < 0.05.

## 3. Results

### 3.1. Apoptogenic Action of CAF + Alcohol

**Figure 1:**
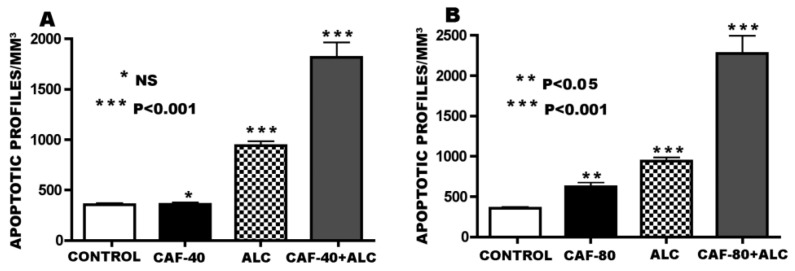
Apoptogenic action of caffeine (CAF) alone or in combination with alcohol. Apoptotic profiles were detected by activated caspase 3 (AC3) immunohistochemical staining. (**A**) Although CAF at 40 mg/kg (CAF-40) caused no increase in apoptosis, when this dose was combined with alcohol, the apoptotic response to alcohol was markedly increased. (**B**) CAF at 80 mg/kg (CAF-80) caused a modest but significant increase in cell death, and when this dose of CAF was combined with alcohol the apoptotic response was dramatically increased.

CAF was administered to P4 infant mice at 40 or 80 mg/kg, and alcohol at 2.5 g/kg, either alone or together with each dose of CAF. The density of apoptotic profiles for each treatment group in the 40 mg/kg CAF experiment is given in Figure 1A and for the 80 mg/kg CAF experiment in Figure 1B. The profile counts for the control group represent the natural rate of neuroapoptosis in the developing mouse brain at this age. The apoptosis counts for the alcohol group in each experiment were significantly increased, and the counts for CAF at the higher dose were increased to a moderate degree, but at the lower dose were not different from the controls. Surprisingly, each dose of CAF, when combined with alcohol, resulted in a much larger increase in neuroapoptosis than could have been predicted by the observed response to CAF alone; the amount of increase was so great that it suggests that a supra-additive (potentiating) mechanism may be operative.

### 3.2. Apoptogenic Action of CAF + NMDA Antagonists (PCP or Ketamine)

**Figure 2:**
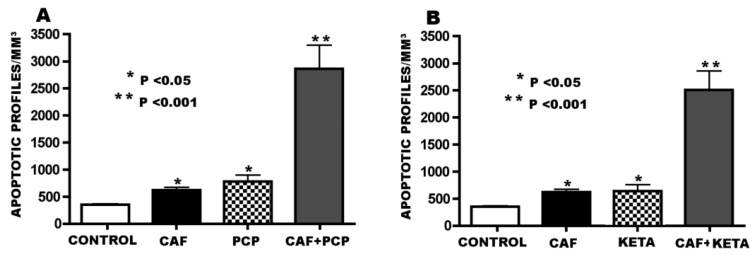
Apoptogenic action of CAF in combination with phencyclidine (PCP) (**A**) or Ketamine (**B**).

CAF was administered to P4 infant mice at 80 mg/kg, and PCP and ketamine were administered at sub-anesthetic doses either alone or in combination with CAF. In Figure 2A,B, apoptosis counts show that both CAF alone and PCP or ketamine alone caused a modest but significant neuroapoptosis response, and when CAF was combined with either drug, there was a much more robust response than could have been predicted by the responses to the individual agents. This indicates that CAF augments neuroapoptosis induced by either of two NMDA antagonist drugs, and the degree of augmentation is in the supra-additive range.

### 3.3. Apoptogenic Action of CAF + GABAmimetics (Diazepam or Isoflurane)

**Figure 3:**
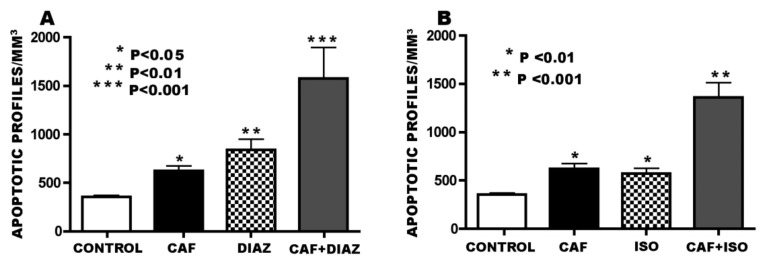
Apoptogenic action of CAF in combination with diazepam (**A**) or isoflurane (**B**).

For the diazepam experiments, CAF was administered at 80 mg/kg and diazepam at 10 mg/kg to P4 infant mice. The histogram in Figure 3A reveals that the neuroapoptosis counts in CAF or diazepam treated mice were significantly increased to a moderate degree and were increased to a much greater degree following CAF + diazepam exposure.

For the isoflurane experiments, CAF was administered at 80 mg/kg and isoflurane was administered as a gas for 2 h at 2% concentration. Because of the respiratory depressant effects of isoflurane and the respiratory stimulatory effects of CAF, this experiment provided an opportunity to observe the interactive effects of these agents on respiration as well as their interactive effects on neuroapoptosis outcome. Exposure of P4 pups to isoflurane alone for 2 h induced respiratory insufficiency in all pups, resulting in dyspnea, cyanosis and 15% mortality. In contrast, there was zero mortality in the control, CAF alone or CAF + isoflurane groups, and none of these groups showed signs of respiratory insufficiency. This signifies that CAF effectively counteracted both the respiratory depressant action of isoflurane and its lethal consequences. The histogram in Figure 3B shows that both CAF and isoflurane increased the neuroapoptosis rate to a moderate but significant degree, and adding CAF to isoflurane resulted in a much greater increase which, like the response to CAF + alcohol or NMDA antagonists, was in a supra-additive range. It is noteworthy that although the isoflurane group experienced significant hypoxia, which might be expected to increase the cell death count, and the CAF + isoflurane group did not experience hypoxia, the exaggerated cell death response was in the CAF + isoflurane group that did not experience hypoxia. 

### 3.4. Overview of CAF’s Pro-Apoptotic Action

**Figure 4:**
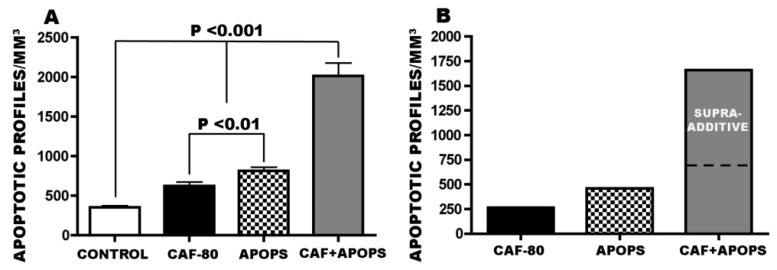
Overview of CAF’s pro-apoptotic action. (**A**) The apoptotic profile count for the composite CAF-80 group and for the composite apoptogens group were both significantly increased compared to the composite control group, and the CAF-80 + apoptogens group was increased significantly and to an extreme degree compared to any of the other three groups. (**B**) The data in A are displayed here with the control values (natural apoptosis) subtracted out so that the remaining values reflect only the amount of cell death attributable to the drug exposures. Adding the bar for CAF-80 to the bar for the apoptogens group would yield a value represented by the dashed line. But the actual value for CAF-80 + apoptogens group was much greater, signifying that CAF acts by a supra-additive mechanism.

The doses selected for each of the primary apoptogens in this study (alcohol, PCP, ketamine, diazepam, isoflurane) were sedating but sub-anesthetic doses that induced a relatively mild neuroapoptosis reaction, and the dose of CAF was one that caused only a relatively mild neuroapoptosis reaction. Consistently, combining CAF with any of these primary apoptogens resulted in a more robust neuroapoptosis response than would have been predicted by the modest responses to the individual agents. In order to provide an overview of CAF’s pro-apoptotic activity in relation to primary apoptogens as a like-acting group of neurotoxic agents, we developed a single composite data set containing the apoptosis density counts for all animals exposed to a primary apoptogen (*n* = 39), a second composite set for all animals exposed to 80 mg/kg CAF (CAF-80) (*n* = 32), a third for all control animals (*n* = 37), and a fourth for all exposed to CAF-80 + an apoptogen (*n* = 37). The histogram in Figure 4A illustrates these composite data. Statistical evaluation by ANOVA revealed a main treatment effect [*F*(3, 136) = 64.00, *P* < 0.001]. By Bonferroni *post hoc* analysis, the mean density count for both the composite apoptogens group and the CAF-80 group was significantly increased compared to the composite control group (*P* < 0.001), and the composite apoptogens group was significantly greater than the Caf-80 group (*P* < 0.01). The density count for the composite CAF-80 + apoptogens group was significantly increased (*P* < 0.001) compared to each of the other three groups. In Figure 4B the composite data for each treatment group have been adjusted to reflect only the density counts attributable to drug treatment (control values representing natural apoptosis subtracted), and this reveals that by an additive mechanism the composite CAF-80 group would add 266.27 profiles/mm^3^ to the composite apoptogens group (dashed line in Figure 4B), but the amount actually added was 1200.98 profiles/mm^3^ (4.5 times the expected amount). This supports the interpretation that CAF exerts a supra-additive pro-apoptotic action when combined with any of several apoptogenic agents.

### 3.5. Regional and Cellular Distribution of CAF’s Pro-Apoptotic Action

**Figure 5:**
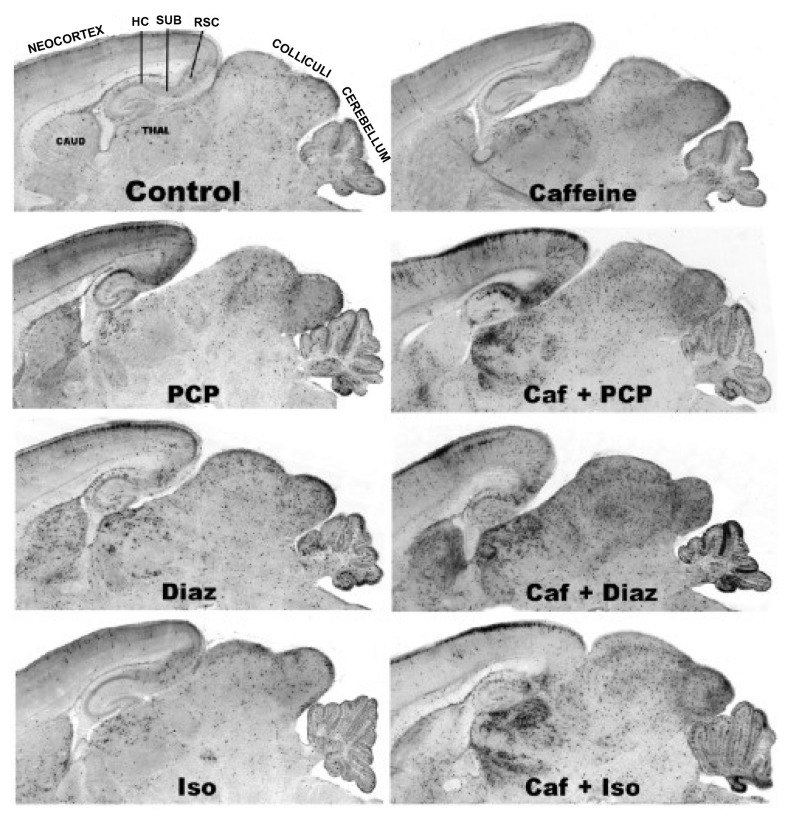
Regional Patterns of Neuroapoptosis. All panels are stained by activated caspase 3 (AC3) immunohistochemistry. In the control brain the AC3-positive profiles (dark specks) are distributed in a relatively sparse and random pattern over many brain regions. In the brains exposed to caffeine, PCP, diazepam (Diaz) or isoflurane (Iso), the density of AC3-positive profiles is moderately increased, especially in neocortex, hippocampus (HC), subiculum (SUB), retrosplenial cortex (RSC), Caudate (CAUD), Thalamus (THAL), colliculi and cerebellum. In the brains exposed to caffeine + PCP, diazepam or isoflurane the density of apoptotic profiles is markedly increased in each of these same regions.

**Figure 6:**
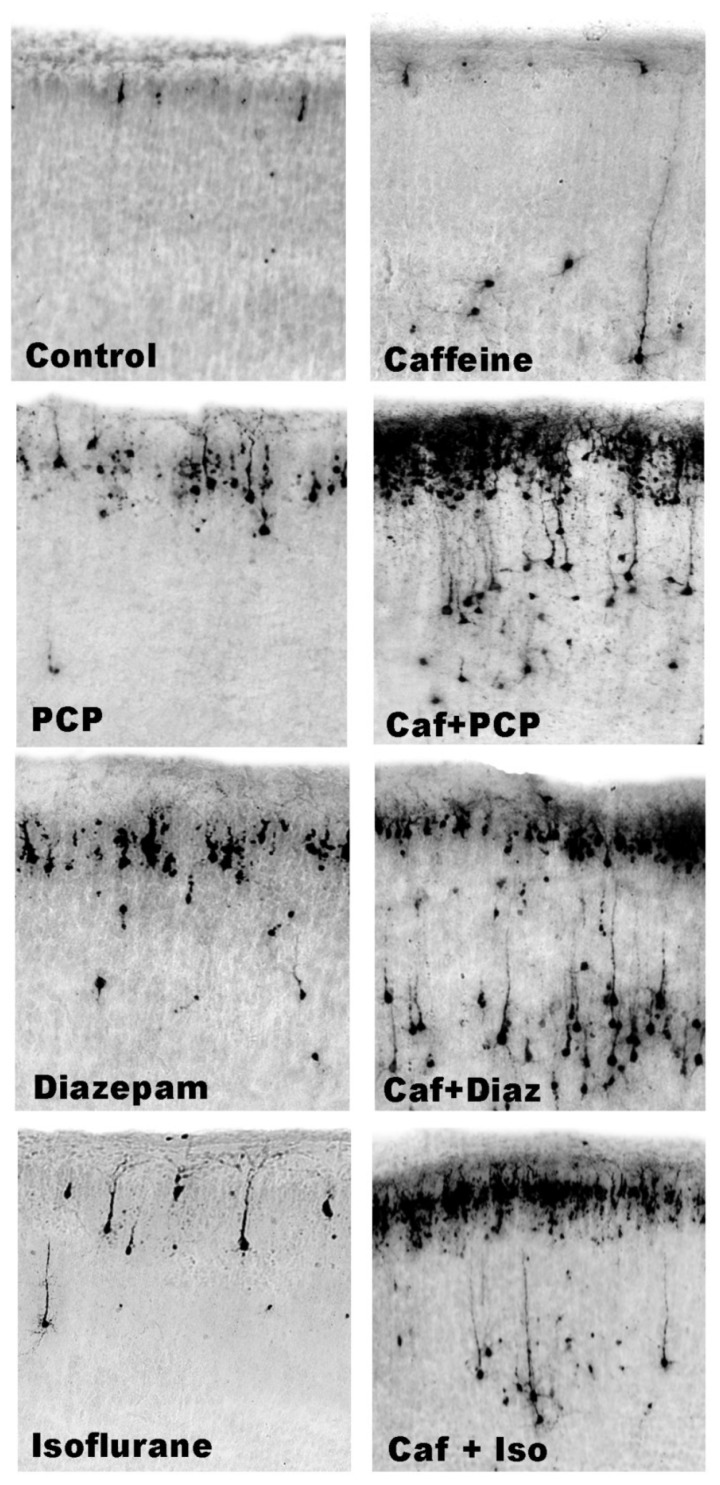
Neuroapoptosis in the Neocortex. These detail views reveal that combining caffeine with PCP, diazepam or isoflurane causes a marked increase in the density of apoptotic profiles in the superficial cortical layers, and also causes spread of the cell death reaction to include the deeper cortical layers.

**Figure 7:**
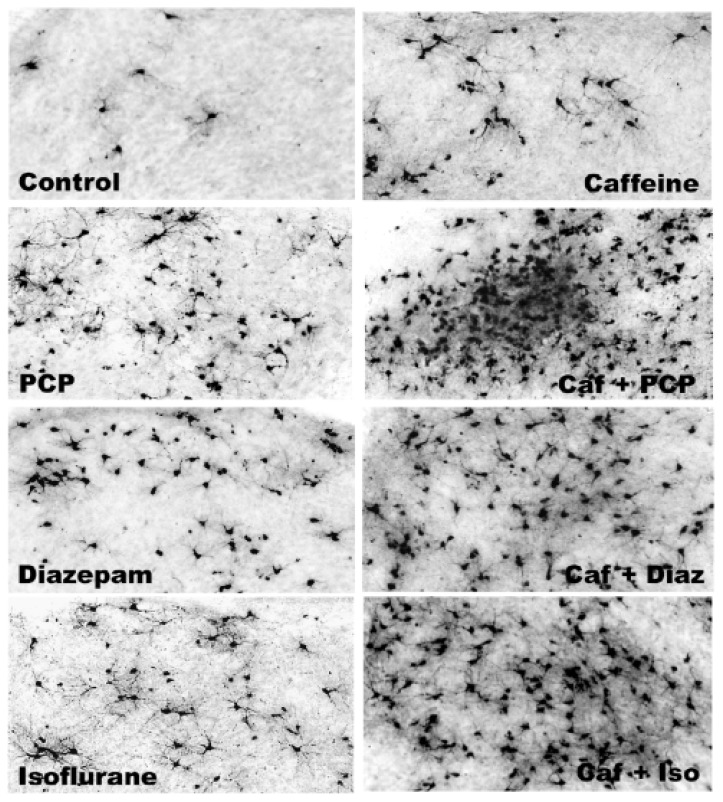
Neuroapoptosis in the Thalamus. These detail views focus on the dorsomedial thalamic nucleus and neighboring thalamic nuclei. Compared to the control brain, there is a substantially higher density of apoptotic profiles in the brains exposed to caffeine, PCP, diazepam or isoflurane, and show a low density of apoptotic profiles in the control brain and a more striking increase in the brains exposed to caffeine in combination with PCP, diazepam or isoflurane.

**Figure 8:**
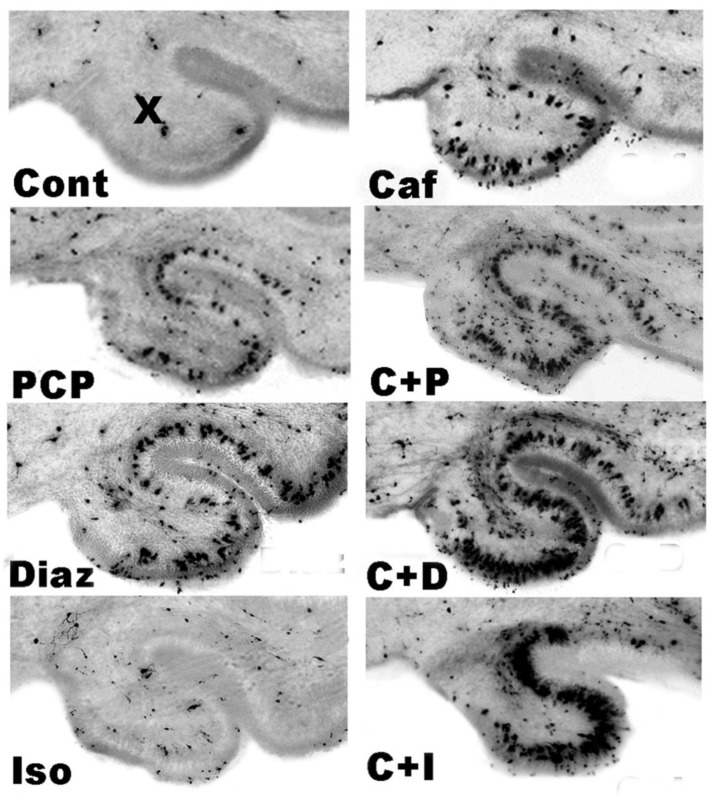
Neuroapoptosis in the cerebellum. These panels illustrate the neuroapoptosis response in folium 10 (X) of the infant mouse cerebellum of a control (Cont) brain, compare to brains exposed to caffeine (Caf), phencyclidine (PCP), caffeine + PCP (C + P), Diazepam (Diaz), caffeine + diazepam (C + D), Isoflurane (Iso) or caffeine + isoflurane (C + I). The large dark profiles in curvilinear display are apoptotic Purkinje cells. The smaller dark profiles are in a migratory status and are of uncertain identity [4].

The regional distribution of apoptotic neurodegeneration induced by NMDA antagonist and GABAmimetic drugs depends on the developmental age at the time of drug exposure. At a given age, both classes of drugs, with some exceptions, tend to affect the same brain regions, although not necessarily the same cell types within a given region. The regions typically affected most severely in the P4 infant mouse are the cerebellum, inferior and superior colliculi, caudate-putamen, many thalamic nuclei, retrosplenial cortex, subiculum and hippocampal formation and all major divisions of the neocortex. The apoptotic profile counts reported herein are given as mean density/mm^3^ across all of these brain regions. The histological appearance of the acute neurodegenerative reaction, as detected by AC3 staining, is depicted for whole brain sagittal sections in Figure 5 and for individual brain regions in Figure 6 (neocortex), Figure 7 (thalamus) and Figure 8 (cerebellum). These illustrations support the interpretation that CAF tends to augment the cell death reaction induced by various primary apoptogens in each of the brain regions where they are typically most active.

### 3.6. Long-Term Neurobehavioral Effects of CAF + PCP or Diazepam

#### 3.6.1. CAF + PCP

**Figure 9:**
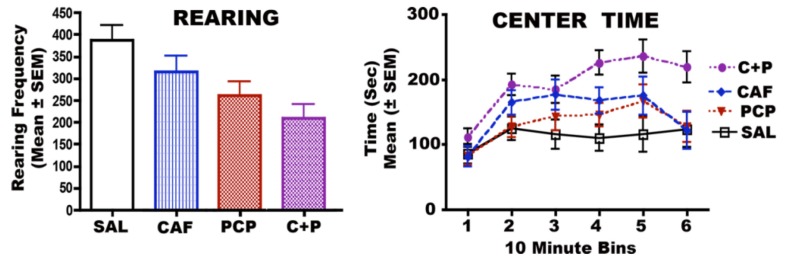
Frequency of Rearing and Time Spent at Center *versus* Edges of Chamber.

When normal mice are placed in a chamber with which they are not familiar, they typically show exploratory behavior, including frequent rearing, sniffing and moving about from one edge of the chamber to another. Mice exposed in infancy to saline, CAF, PCP or CAF + PCP were evaluated as adolescents for these behaviors when placed in an activity chamber (representing a novel environment). The data for frequency of rearing and the amount of time spent in the center of the chamber *versus* at the edges of the chamber are documented in Figure 9. Compared to controls, frequency of rearing was moderately decreased in CAF-exposed mice (non-significant), and was decreased to a greater degree in the PCP-exposed group (*P* < 0.01), and to even a greater degree in the CAF + PCP exposed group (*P* < 0.0006). Time spent in the center of the chamber and away from the edges was also increased to an exaggerated degree in CAF + PCP mice compared to all other groups (*P* < 0.0007). Decreased rearing and reduced exploratory behavior are consistent with an attention deficit disorder [72], while staying in the center of the chamber and avoidance of the edges is a sign of increased emotionality and anxiety. 

**Figure 10:**
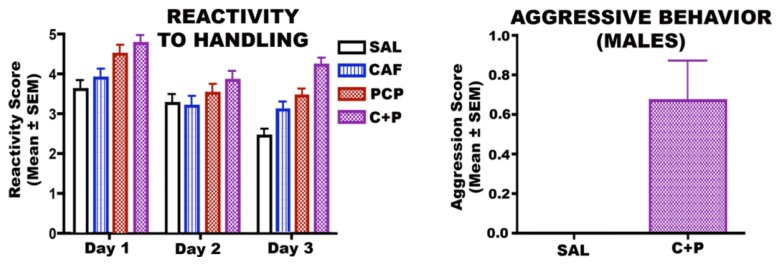
Reactivity to Handling and Male Aggression.

Because the PCP and CAF + PCP mice showed signs of irritability and agressiveness, we performed a reactivity to handling test and subjected the saline and CAF + PCP males to a resident intruder test that measures aggressivity. These results (Figure 10) show that on three successive days of testing the PCP, and much more so the CAF + PCP mice, displayed hyper-reactivity to handling and, whereas the saline controls became habituated to handling by the 3rd day of testing, the CAF + PCP mice failed to habituate. In the resident intruder test, the CAF + PCP mice often attacked and initiated fights with other males, whereas the saline control males showed no aggressive tendencies at all. The caffeine, PCP and caffeine + PCP mice were also tested on the Morris water maze and showed no deficits in learning/memory domains (data not shown).

#### 3.6.2. CAF + Diazepam

**Figure 11:**
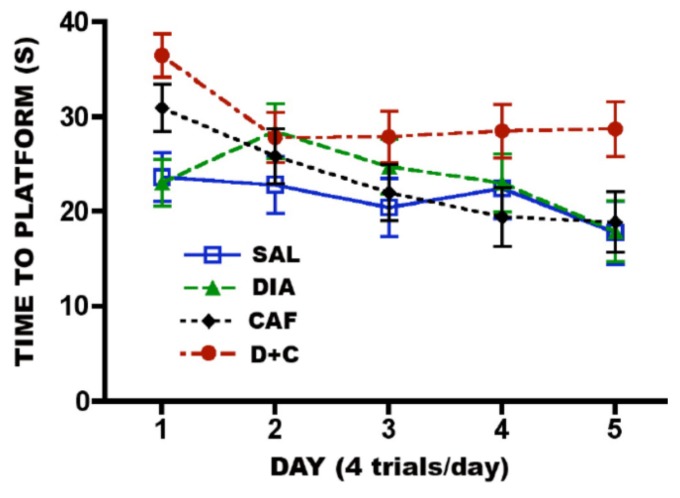
Latency Scores: Morris Water Maze.

In diazepam-treated mice, adding CAF did not result in significant differences in measures of reactivity to handling or open field activity, but did impair learning. In the Morris Water Maze there were no differences between groups in swim speeds or cued trials, but distance traveled and latency to find the platform were significantly increased in diazepam + CAF mice and this group showed no evidence of learning across the 5 days of testing (Figure 11).

## 4. Discussion

CAF enjoys the reputation of being a highly beneficial drug for use in neonatal medicine. By blocking adenosine receptors, CAF is believed to increase the release of glutamate at excitatory receptors and, therefore, is used to stimulate respiration in premature infants who are apnea-prone due to weakness of the respiratory reflex. Among the benefits recently attributed to CAF therapy for premature infants is decreased morbidity associated with bronchopulmonary dysplasia and cerebral palsy [65]. In addition to these potential benefits, neonatologists currently consider CAF so safe that they are questioning the need for monitoring CAF blood levels when administering it semi-chronically to premature infants [62,63,73].

The present study was a proof of concept study designed to determine whether CAF has pro-apoptotic properties in the developing brain, which might cause it to interact unfavorably with other drugs that have pro-apoptotic properties. Our findings document that CAF does have pro-apoptotic actions in the developing brain, and when administered in combination with other agents that have neuroapoptogenic properties it can cause a striking potentiation of their apoptogenic activity. Thus, notwithstanding CAF’s potential benefits, our data suggest that it may also, under certain circumstances, be harmful for the developing brain. The two human settings in which substantial CAF exposure occurs and might potentially be harmful are: (1) A drug abuse setting in which a pregnant mother drinks coffee or other CAF-containing beverages or foods while abusing drugs that have apoptogenic properties (e.g., alcohol, PCP, ketamine, benzodiazepines or barbiturates). (2) A medical setting in which CAF is administered to premature infants as a respiratory stimulant when these infants are also being exposed to sedative/anesthetic drugs. In the following paragraphs, we will discuss risk associated with CAF exposure in each of these settings.

Drug Abuse Setting: Our findings indicate that CAF has the potential to increase the neurotoxicity of several drugs of abuse (DOA), including those that act at NMDA receptors, those that act at GABA receptors, and alcohol, which interacts with both of these receptor systems. To further assess the human relevance of these findings it will be necessary to conduct dose/response testing to determine threshold doses of CAF and each DOA for triggering a potentiated apoptotic response. In the absence of such data, it is unclear whether CAF at doses typically ingested by pregnant women in combination with apoptogenic DOA at doses in the abuse range would trigger a potentiated neuroapoptosis reaction. Risk would presumably be greatest for CAF in combination with alcohol, because millions of fetuses annually throughout the world are exposed to alcohol, sometimes under binge conditions [74,75], and a high percentage of those fetuses are likely to be exposed simultaneously to CAF, given the high frequency with which CAF is used and abused in modern societies [76]. Factors to consider in evaluating risk posed by CAF/alcohol exposures are as follows: (1) A single exposure to alcohol, triggers widespread neuroapoptosis in the developing brain of infant rodents [2,7] or fetal monkeys [35,36]; (2) Binge drinking [75], even on a single occasion [74] during pregnancy, is associated with long-term neurobehavioral disturbances in the offspring; (3) Pregnant mothers with a strong alcohol and strong CAF habit may expose their fetuses to binge amounts of alcohol [74,75] and repetitive boluses of CAF intake [77] throughout a given day, and on multiple occasions during a given pregnancy; (4) During the last trimester of pregnancy, the half-life for CAF in maternal blood is 2–3 times greater than in the non-pregnant state [78]; (5) Even in the non-pregnant state, alcohol inhibits the liver enzyme system that catalyzes the metabolism of CAF, thereby prolonging its half life [79]. 

We studied the long-term neurobehavioral effects of CAF in combination with the NMDA antagonist DOA, PCP, and found that both CAF and PCP caused an increase in AD/HD-like behaviors, and the two drugs in combination caused a more pronounced AD/HD-like syndrome. This suggests a special relationship between CAF, the NMDA receptor system and AD/HD-like behavioral disorders, which is consistent with other prior reports [80,81], including the observations of Fredriksson *et al*. [41] who found that treatment of 10 day old rats with ketamine (NMDA antagonist DOA and widely used general anesthetic) caused long-term AD/HD-like behavioral disturbances. We also assessed the long-term neurobehavioral effects of CAF in combination with the GABA agonist, diazepam, and found that the CAF/diazepam combination caused learning/memory impairment. There was no cross-over between the diazepam-linked and PCP-linked syndromes in that CAF/diazepam did not cause AD/HD-like disturbances, and CAF/PCP did not cause learning impairment. It will be of interest to study this phenomenon further with an aim toward establishing correlations between patterns of neuronal losses caused by a given drug combination, and neurobehavioral outcomes associated with that drug combination. It will also be of interest to do additional testing to determine whether other NMDA antagonists, in combination with CAF, preferentially cause AD/HD-like behavioral syndromes, and other GABA agonists, in combination with CAF, preferentially cause learning disability syndromes. 

Medical setting: CAF exposure of premature infants is potentially of concern because the duration of exposure is typically quite prolonged (continuous for days or weeks), and the doses of CAF used are sometimes quite high, based on the current belief that CAF is safe for premature infants even at high doses [62]. The doses of CAF used in our experiments produce blood CAF levels in the infant mouse that are in the same range as those considered safe for human premature infants. The doses of sedatives or anesthetics used in our experiments are doses required for the degree of sedation that human premature infants experience when they are subjected to procedural sedation or surgical anesthesia, except that procedural sedation sometimes involves continuous or repeated exposure over a period of days or weeks, and in our study mice were exposed on only a single occasion. While extrapolation from rodents to humans is always fraught with uncertainties, the original evidence documenting apoptogenicity of sedative and anesthetic drugs [1,2,3,6] was generated in infant rodents, then was reproduced in infant and fetal non-human primates [28,29,30,31,32,33,34,35,37,38,39,40], and there now are seven recently published studies [53,54,55,56,57,58,59] documenting that brief exposure of human infants to anesthesia is associated with increased risk for long-term neurobehavioral disturbances, including disturbances relevant to both AD/HD and learning disability domains. While some of the studies [55,56,57] have been interpreted as evidence that it may require multiple exposures or a total exposure duration ≥2 h for a significant neurocognitive disability effect, other studies [53,54,58,59] support the interpretation that a single exposure to anesthesia for less than 2 h is sufficient to increase the risk for neurocognitive impairment. Collectively, these recent developments suggest that, for this type of toxic mechanism, rodent data may be reliable predictors of human risk.

It may seem like a contradiction that CAF could promote widespread apoptotic cell death throughout the brain and also be protective against cerebral palsy. However, these are not mutually exclusive possibilities. Protection against cerebral palsy can be explained by CAF preventing apnea and hypoxia which, if extreme, can cause pathological accumulation of glutamate at excitatory receptors, leading to excitotoxic neurodegeneration, whereas potentiation of anesthesia-induced neuroapoptosis is due to the inherent pro-apoptotic property of CAF (mechanism unknown), and a comparative analysis of excitotoxic *versus* apoptotic cell death reveals that these are two separate and distinct forms of cell death [12,67]. In fact, excitotoxic neurodegeneration in the infant rodent brain is triggered by excessive activation of NMDA receptors [82,83], and apoptotic neurodegeneration is triggered by insufficient activation of NMDA receptors [1,83]. In our present experiments, mice exposed to isoflurane alone for 2 h experienced a severe and potentially lethal degree of cerebral hypoxia. Interestingly, these isoflurane-exposed mice displayed only a moderate degree of neurodegeneration, all of which can be explained by the apoptogenic action of isoflurane, and none of which had hypoxia-like excitotoxic characteristics. Moreover, adding CAF to the isoflurane protocol prevented isoflurane from inducing cerebral hypoxia, while causing it to trigger a much more severe neuroapoptosis reaction. This provides cogent new evidence supporting the interpretation that hypoxic-ischemic (excitotoxic) neurodegeneration and apoptotic neurodegeneration are two different forms of cell death, and factors that promote (or inhibit) excitotoxicity in the developing brain can potentially have an opposite effect on apoptogenicity.

That CAF has pro-apoptotic properties and can induce neuroapoptosis in the developing brain is not a novel observation. One decade ago, Kang *et al*. [66] demonstrated that CAF induces widespread apoptotic neurodegeneration in the *in vivo* infant rat brain, and also induces apoptotic death of cultured neurons *in vitro*. The novel finding that we are adding to the picture is that CAF markedly potentiates the neuroapoptogenic action of other drugs, including alcohol and many drugs used widely in pediatric and obstetric medicine. We are not aware of any studies on premature infants designed to rule out a neurotoxic action of CAF in combination with anesthetic drugs. The widely cited multicenter study by Schmidt *et al*. [65] in which it was reported that CAF is protective against cerebral palsy, used a maximum CAF loading dose of 20 mg/kg which is 1/4 as high as the maximal doses that currently are being used in some NICUs and are considered safe [62,63,73]. Moreover, in the Schmidt *et al.* study there is no mention of anesthesia exposure for either the group receiving CAF or the group receiving placebo. Therefore, it is entirely possible that low blood levels of CAF with minimal anesthesia exposure is protective against cerebral palsy, and high blood levels of CAF with substantial anesthesia exposure may be detrimental. 

It is widely recognized that children with a history of premature birth and prolonged care in the NICU, have a high incidence of neurocognitive impairment [84], but drugs used in the NICU for procedural sedation or to stimulate respiration have, thus far, escaped scrutiny as potential contributory factors. Our findings indicate the need for new research aimed at clarifying the degree of risk posed by combined exposure of premature infants to CAF and sedative/anesthetic drugs, and a parallel program aimed at learning how to take full advantage of CAF’s beneficial effects while avoiding its potentially harmful effects. In view of evidence that morbidity associated with cerebral palsy is reduced by CAF administration at a low dose [65], a simple measure that might be taken, sooner better than later, would be to set limits on the dose of CAF that is used in the NICU. Implementing this recommendation could be highly beneficial and is not likely to be detrimental, although clearly it challenges the growing belief that CAF is safe for premature infants over a wide range of doses [62,63,73]. 

## 5. Conclusions

Our data suggest the need to consider whether CAF may have harmful as well as beneficial effects on the developing brain, and the need for research aimed at taking advantage of its beneficial effects while avoiding its potentially harmful effects.
